# Microvascular Obstruction in ST-Segment Elevation Myocardial Infarction: Looking Back to Move Forward. Focus on CMR

**DOI:** 10.3390/jcm8111805

**Published:** 2019-10-28

**Authors:** Cesar Rios-Navarro, Victor Marcos-Garces, Antoni Bayes-Genis, Oliver Husser, Julio Nuñez, Vicente Bodi

**Affiliations:** 1 Institute of Health Research INCLIVA, 46010 Valencia, Spain; cesar_rios1@hotmail.com (C.R.-N.); yulnunez@gmail.com (J.N.); 2 Cardiology Department, Hospital Clínico Universitario, 46010 Valencia, Spain; vic_mg_cs@hotmail.com; 3 Centro de Investigación Biomédica en Red-Cardiovascular (CIBER-CV), 28029 Madrid, Spain; abayesgenis@gmail.com; 4 Cardiology Department and Heart Failure Unit, Hospital Universitari Germans Trias i Pujol (Badalona) and Department of Medicine Universitat Autonoma de Barcelona, 08916 Barcelona, Spain; 5 Department of Cardiology, St-Johannes Hospital, 44137 Dortmund, Germany; oliver.husser@gmail.com; 6 Department of Medicine, Universidad de Valencia, 46010 Valencia, Spain

**Keywords:** myocardial infarction, microvascular obstruction, reperfusion injury

## Abstract

After a myocardial infarction (MI), despite the resolution of the coronary occlusion, the deterioration of myocardial perfusion persists in a considerable number of patients. This phenomenon is known as microvascular obstruction (MVO). Initially, the focus was placed on re-establishing blood flow in the epicardial artery. Then, the observation that MVO has profound negative structural and prognostic repercussions revived interest in microcirculation. In the near future, the availability of co-adjuvant therapies (beyond timely coronary reperfusion) aimed at preventing, minimizing, and repairing MVOs and finding convincing answers to questions regarding what, when, how, and where to administer these therapies will be of utmost importance. The objective of this work is to review the state-of-the-art concepts on pathophysiology, diagnostic methods, and structural and clinical implications of MVOs in patients with ST-segment elevation MIs. Based on this knowledge we discuss previously-tested and future opportunities for the prevention and repair of MVO.

## 1. Introduction

Just one century ago, James B. Herrick reported the first human evidence regarding the role of thrombotic obstruction of coronary arteries in the pathophysiology of myocardial infarction (MI), including the observation that this syndrome did not necessarily imply the immediate death of patients [1]. Since then, numerous milestones have been passed. Nowadays, the routine use of coronary reperfusion has dramatically reduced acute mortality in patients with ST-segment elevation MIs (STEMIs) [2]. Nevertheless, despite successful reperfusion at the epicardial level, the deterioration of myocardial perfusion persists in a considerable number of patients. This phenomenon is known as microvascular obstruction (MVO) and can occur in 50% to 60% of cases [3] and has been associated with adverse ventricular remodeling and a heightened risk of future cardiovascular events [4,5]. MVO was first described in experimental models by Krug in the 60s, and the concept was defined by Kloner in the 70s [6]. Unfortunately, 50 years after these contributions, MVO remains an unresolved problem that continues to have deleterious structural and prognostic consequences [5,7,8].

Further knowledge on the basic mechanisms, diagnostic tools, and implications of MVO is crucial to achieving prevention and exploring potential repair opportunities. Ultimately, this process will help overcome one of the last barriers in the management of STEMI and to further reduce its associated mortality.

## 2. Pathophysiology

**Figure 1:**
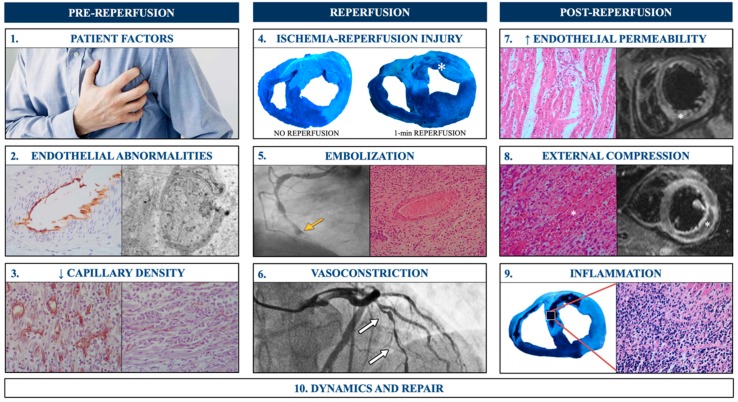
Mechanisms implicated in the pathophysiology of microvascular obstruction (MVO) after myocardial infarction. MVO is a multifactorial phenomenon caused by the interaction of a variety of mechanisms that act predominantly (but not only) at: Pre-reperfusion: (**1**) Patient factors. (**2**) Endothelial abnormalities. Optic (left) and electronic (right) microscopic views show diffuse microvascular endothelial damage prior to reperfusion. (**3**) Decreased capillary density in areas with MVO soon after ischemia onset. Reperfusion: (**4**) Ischemia-reperfusion injury. After 90 min ischemia in a swine model, MVO (asterisk) was macroscopically undetectable if the artery remained occluded (left) but was clearly present 1 min after reperfusion (right). (**5**) Embolization. Distal migration of thrombotic material (arrow) after primary angioplasty. (**6**) Vasoconstriction. Severe vasospasm (arrows) immediately after reperfusion. Post-reperfusion. (**7**) Increase in endothelial permeability. Hematoxylin-eosin staining in experimental samples (left) and T2-weighted cardiovascular magnetic resonance in patients (right) display the consequences of increased endothelial permeability; namely, edema (asterisk) and hemorrhaging. (**8**) External compression. Severe hemorrhage (asterisk) at the microscopic (left) and macroscopic (right) levels contribute to microvascular compression and MVO. (**9**) Inflammation. Massive inflammatory reaction in the core of an area with severe MVO. (**10**) Dynamics and repair are crucial to understanding the pathophysiology of MVO. See Section 6 for further details on this topic.

A number of underlying mechanisms act both simultaneously and sequentially in the complex and not yet fully understood pathophysiology of MVO. The 10 most relevant factors and the phase when they predominantly occur are briefly discussed below (Figure 1).

### 2.1. Predominantly Prior to Reperfusion

#### 2.1.1. Patient Factors

The presence of a large at-risk area (extensive ECG abnormalities, heart failure upon presentation, or delayed reperfusion) or previously damaged microcirculation (diabetes mellitus or delayed reperfusion) predicts MVO’s occurrence [3].

#### 2.1.2. Endothelial Abnormalities

After prolonged ischemia, endothelial cells from arteries to coronary capillaries suffer functional and structural damage that worsens with reperfusion and contributes to the development of MVO [9].

#### 2.1.3. Decrease in Capillary Density

MVO is in part driven by the loss of small vessels that starts shortly after the onset of ischemia and sharply accelerates at the time of reperfusion [10].

### 2.2. Predominantly at Reperfusion

#### 2.2.1. Ischemia-Reperfusion Injury

**Figure 2:**
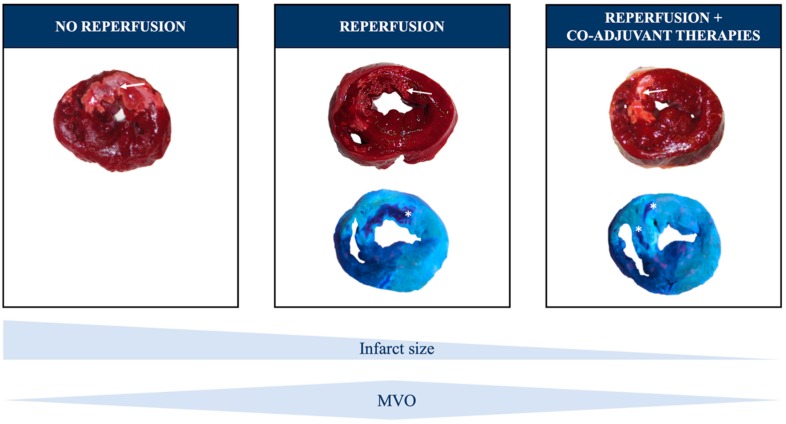
Coronary reperfusion and microvascular obstruction (MVO). The absence of coronary reperfusion leads to transmural infarction (left panel). A complete and prompt reperfusion of the culprit artery, ideally by percutaneous coronary intervention, reduces infarct size but may imply a certain ischemia-reperfusion injury, including MVO (central panel). Hopefully, future co-adjuvant therapies beyond reperfusion will further diminish infarct size and MVO extent, and consequently, will improve patient outcomes (right panel). Arrows point at areas with infarction and asterisks at regions with MVO.

Although early reperfusion is necessary, experimental and clinical studies demonstrate its association with certain hemodynamic and oxidative stresses on coronary circulation, leading to MVO [11] (Figure 2).

#### 2.2.2. Embolization

Micro-embolization is particularly frequent in the context of primary percutaneous intervention due to the manipulation of unstable plaques with important thrombotic burden [3,12].

#### 2.2.3. Vasoconstriction

Both ischemia, and subsequently, reperfusion injuries, can enhance endothelium-derived vasoconstrictor products and may hamper myocardial perfusion [3,13].

### 2.3. Predominantly Post-Reperfusion

#### 2.3.1. Increase in Endothelial Permeability

Reperfusion injury includes sudden swelling and the disruption of endothelial cells that facilitate capillary obstruction; extravasation of the blood content into the interstitium; and intra-myocardial hemorrhaging [3,13].

#### 2.3.2. External Compression

External compression of the microvasculature by hemorrhage and myocardial edema decisively contributes to MVO [13,14].

#### 2.3.3. Inflammation

After MI, inflammation is a physiological and necessary response that initiates repair. However, when out of control, this process can lead to unnecessary myocardial and microvascular damage [15,16]. Neutrophils, via aggregation and formation of neutrophil extracellular traps, decisively participate in MVO progression [17].

### 2.4. From the Onset of Ischemia until Late after Reperfusion

#### Dynamics and Repair

MVO is a highly dynamic process throughout onset, expansion, and repair. A specific chapter is dedicated to this issue at the end of the manuscript.

In summary, MVO pathophysiology is complex and multifactorial, and while significant progress has been made over recent decades, a sustained research effort is necessary to fully elucidate this process. Ultimately, this will help us obtain and apply effective preventive and reparative therapies.

## 3. Diagnostic Tools

**Figure 3:**
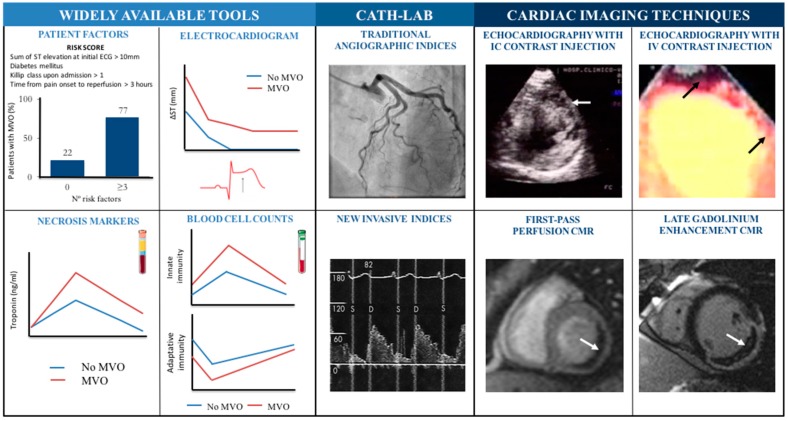
Diagnostic tools to detect the presence of microvascular obstruction (MVO) in patients with ST-segment elevation myocardial infarctions. The identification of MVOs can be accomplished using different tools, classified as: widely available tools (left); catheterization lab (central): traditional angiographic indices (TIMI and TIMI frame count indices, “TIMI myocardial perfusion grade”, or “myocardial blush grade”) and new invasive indices (reserve of coronary flow velocity, the diastolic deceleration time of coronary flow velocity, or the presence of systolic flow inversion); and cardiac imaging techniques (right). See text (Section on “diagnostic tools”) for further information on each specific item. Arrows point at areas with MVO. Innate immunity cells are considered neutrophils and monocytes, whereas adaptative immunity is comprised of lymphocytes and eosinophils. CMR = cardiovascular magnetic resonance; IC = intracoronary; IV = Intravenous; PCI = primary coronary intervention; TIMI = thrombolysis in myocardial infarction.

In STEMI, if the recovered coronary flow is not complete as determined by angiography (grade “thrombolysis in myocardial infarction” (TIMI) 3 flow), severe structural damage can occur. Furthermore, MVO occurs in at least 30% of the cases with TIMI 3 flow, and as a consequence, extensive infarctions and adverse ventricular dilatation take place [7]. Reliable diagnostic tools to accurately assess myocardial perfusion are of utmost importance. Some of them are widely available, while others are obtained in the catheterization lab or using cardiac imaging techniques (Figure 3 illustrates all tools discussed below).

### 3.1. Widely Available Tools

Certain baseline characteristics are of great value in foreseeing the risk of MVO occurrence upon patient arrival. To this end, our group developed a simple scoring method which includes four predisposing factors: Killip class upon admission > 1, time from pain onset to reperfusion > 3 hours, diabetes mellitus, and sum of ST-segment elevation at the initial ECG > 10 mm. The risk of displaying at least one segment with MVO in CMR images ranges from 22% if none are present to 77% if three or more factors are present [18] (Figure 3).

The electrocardiogram represents a cost-effective and widely-available tool. The presence of any residual ST-segment elevation in Q-leads is a powerful predictor of microvascular damage [19].

In the field of biomarkers, an important and early rise in troponin is associated with more extensive MVO [20]. Regarding white blood cells, increases in innate immunity cell counts (neutrophils and monocytes) and decreases in adaptive-effector immunity cell counts (lymphocytes and eosinophils) are associated with MVO [15,16].

### 3.2. In the Catheterization Lab

Once in the catheterization lab, certain traditional angiographic indices, such as the filling rate in the culprit artery (TIMI and TIMI frame count indices) and myocardial contrast uptake (“TIMI myocardial perfusion grade” and “myocardial blush grade” indices), provide an initial estimation of myocardial perfusion, but normal values do not guarantee preserved perfusion and are subject to high inter-observer variability. Recently, new invasive indices based on flow (such as the reserve of coronary flow velocity, the diastolic deceleration time of coronary flow velocity, or the presence of systolic flow inversion) and resistance (such as indices of microvascular resistance or coronary pressure at zero flow) are being investigated [3,13]. These indicators can improve the accuracy of traditional angiographic indices, although wider validation studies will be necessary before recommending their routine use.

All the parameters discussed thus far are either widely available or immediate. However, they are proxies that do not definitively confirm or rule out the presence and extent of microvascular damage. Recent progress in diagnosis has been mainly based on advances in two non-invasive imaging techniques: contrast echocardiography and cardiovascular magnetic resonance (CMR).

### 3.3. Cardiac Imaging Techniques

The combined use of transthoracic echocardiography with intracoronary ultrasound contrast injections provided the first indisputable clinical evidence that, even after re-establishing TIMI 3 flow, the lack of myocardial contrast uptake has deleterious consequences for patients. This technique was replaced, with certain limitations in terms of image quality and reproducibility, by the use of echocardiography with intravenous contrast injections [3,13].

In this context, the incorporation of CMR was decisive, now representing the gold-standard non-invasive technique for the comprehensive assessment of the structural consequences of STEMI [7,18]. More than a decade ago, our group and others pointed out that CMR was the most reliable approach for studying MVO and its consequences [3,21,22].

Contrast CMR assesses the state of microcirculation in two different fashions. Delayed arrival of contrast into the infarcted area during its first pass (within the first minute after intravenous contrast administration) strongly suggests microvascular damage. This index has been criticized for its excessive sensitivity since it appears altered in the vast majority of STEMI patients. Currently, the most widely-used method to diagnose and quantify MVO is based on the analysis of late gadolinium enhancement [3,22,23]. A few minutes after injection, gadolinium vanishes from the myocardium. The persistence of contrast uptake allows an accurate delineation of the hyper-enhanced infarcted area. MVO was defined as lack of contrast in the core of an infarcted, hyper-enhanced area and can bring about deleterious structural and prognostic consequences for STEMI patients [7,22,23].

To summarize, the last two decades have seen important progress in the detection of MVO from purely clinical predictors, then through widely available markers and invasive parameters, and onto the advent of sophisticated non-invasive imaging techniques. Currently, late gadolinium enhancement CMR is the most reliable technique for determining the presence and magnitude of structural and clinical implications of MVO.

## 4. Clinical and Structural Implications

**Figure 4:**
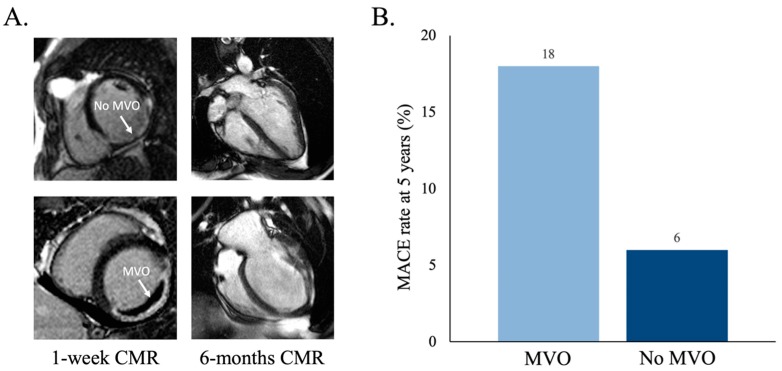
Clinical and structural consequences of microvascular obstruction (MVO) as derived from cardiovascular magnetic resonance (CMR) in ST-segment elevation myocardial infarction patients. (**A**) A CMR after 6 months, showing that more severe left ventricular remodeling and systolic deterioration occurs in patients with MVO (lower panel) compared with those without MVO (upper panel) at 1-week CMR (adapted from 7). (**B**) Patients with MVO at pre-discharge display a higher rate of major adverse cardiac events (MACE; death, re-infarction, or re-admission for heart failure) during follow-up than those without MVO (updated from [24]).

The presence and magnitude of MVO strongly determines the resulting structural damage and survivorship of STEMI patients [4,24] (Figure 4). Single-center and pooled analyses of registries using CMR soon after STEMI have fueled this concept [5,6]. In the era of generalized reperfusion, CMR has revealed a surprisingly high prevalence of MVO (up to 50%–60% of patients). Its deleterious structural effects in terms of more extensive infarct size, more depressed systolic function, less contractile recovery, more adverse remodeling, right ventricular repercussion, or the occurrence of ventricular thrombi has been comprehensively demonstrated [3,7,24,25].

The verification of such a negative structural impact of MVO motivated huge interest in exploring its prognostic implications. Results derived from prospective registers and meta-analyses have been conclusive: MVO constitutes a strong short and long-term prognostic marker for major adverse cardiac events after STEMI [4,8,23]. In a recent meta-analysis, the presence of MVO independently multiplied the two-year risk of death, re-infarction, or heart failure by 3.7 [21].

Thus, in recent years, extraordinary progress has been made in understanding the pathophysiology and diagnosis of MVO, as well as its crucial role in the structural and clinical outcomes after STEMI. Prevention and repair of MVO represent the next (and maybe the last) challenges to overcome.

## 5. Therapeutic Options. Past, Present, and Future

Over the years, promising results have been obtained in highly-controlled experimental models or in small groups of patients. Nevertheless, although some beneficial effects on surrogate endpoints have been observed (Table 1), none of these studies have consistently reduced the extent of CMR-derived MVO and effected a significant decline in clinical events. Table 1 displays the main therapeutic options tested to date based on the previously-mentioned underlying pathophysiological mechanisms of MVO.

A number of factors may explain this lack of translation [72]. While experimental models can prove the pathophysiological role of a specific pathway under highly controlled conditions, they do not necessarily reflect the multifactorial milieu of MVO in patients. Avidity to obtain positive results, lack of interest in publishing negative results, and authors’ self-censorship have probably led to the rapid dissemination of optimistic messages insufficiently supported by clinical evidence. Eventually, this may discourage researchers from attempting to culminate their investigations into effective clinical therapies [73].

Moreover, the complexity of MVO strongly suggests the need for a combined approach, addressing all or part of the myriad of pathophysiological mechanisms in play. However, which therapies should be included in such a multidisciplinary strategy is currently not known [3,73].

At present, in order to avoid frustration, we need to look at the great advances implemented in the last few years to prevent or minimize MVO, and focus on further research in the arduous journey toward defeating MVO (graphical abstract). In fact, education (from childhood) and primary prevention of cardiovascular risk factors, along with reinforcement of the current management of STEMI focused on prompt coronary reperfusion, quantitatively represent, by far, the most effective and rewarding measures in preventing and minimizing the burden of MI, and subsequently, of MVO [74]. A short part of this journey specifically addressed to overcoming the deleterious effects of already-established MVO remains unfinished. Sustained research efforts into co-adjuvant therapies beyond reperfusion are needed to complete this endeavor (graphical abstract).

**Figure 5:**
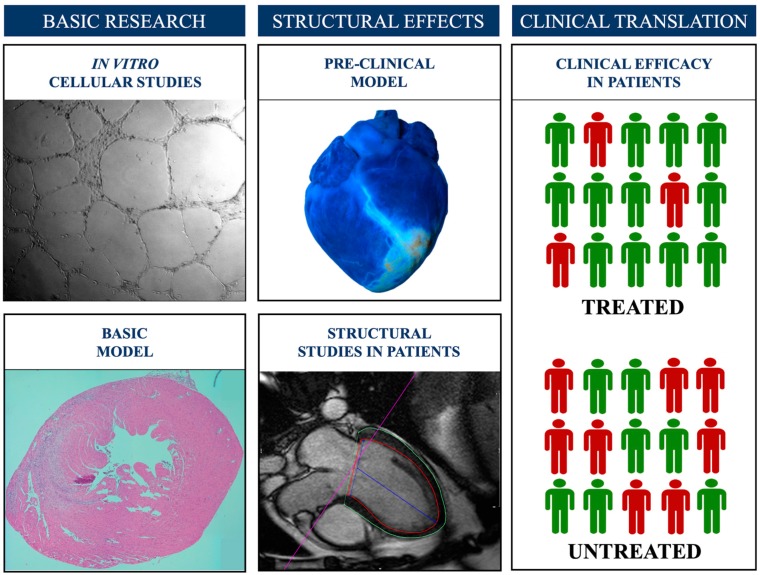
The proposed research steps in the development of novel therapeutic opportunities to prevent, minimize, or repair microvascular obstruction following myocardial infarction.

In our view, future studies addressing the effects of novel co-adjuvant therapies beyond early reperfusion should follow a series of rigorous premises from bench to bedside [75] (Figure 5).

Firstly, the beneficial effects of such therapies must be demonstrated in basic and highly controlled models, either in in vitro cellular studies or in small animal models (such as mice). Secondly, basic approaches with the possibility of translation need prior validation in experimental models mimicking the clinical scenario, such as the infarction-reperfusion swine model. Thirdly, if results obtained in basic research deserve to be analyzed in patients, trials focused on safety and assessing the eventual structural benefit in STEMI patients should be the first step in clinical research. For homogeneity, studies in this phase would include patients, first those with STEMI, those at high risk of MVO (i.e., with anterior infarction due to proximal disease), and those with a time of ischemia not too short (where co-adjuvant therapies probably are not necessary) nor too long (when damage is irreversible). The effects should be independently quantified using the reference technique for this purpose; namely, CMR.

If the new approach is to be considered effective, validation in large, multi-center clinical trials with the prospective and consecutive inclusion of patients focused not only on the reduction of mortality but also on the prevention of long-term heart failure would constitute the last and necessary step prior to recommending its routine use in clinical practice.

Although arduous, this process is the best path toward eventually transforming some of the achievements already obtained by basic research into routine benefits for our patients. So far, the use of intravenous metoprolol prior to reperfusion is the only approach that has almost completed this strategy [63]. The effects of the drug in vitro and in experimental models have been demonstrated. To a lesser extent, CMR-derived MVO has been reported in treated patients. The last step, reduction of clinical events in clinical trials, is still pending. On the contrary, other approaches such as cyclosporin A, were demonstrated to reduce MVO extension in an experimental swine MI model [76], but these results were not confirmed in multicenter clinical trials [77,78].

## 6. Understanding the Dynamics and Repair of MVO for Addressing WH-Questions on Therapy

**Figure 6:**
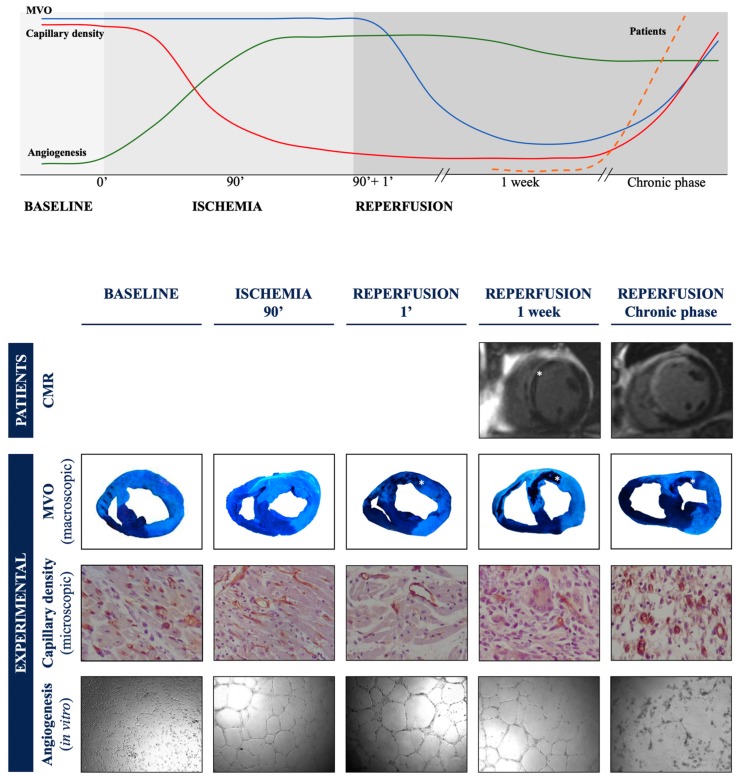
Dynamics of occurrence and repair of microvascular obstruction (MVO). Asterisks point to areas with MVO (adapted from [10]).

**Figure 7:**
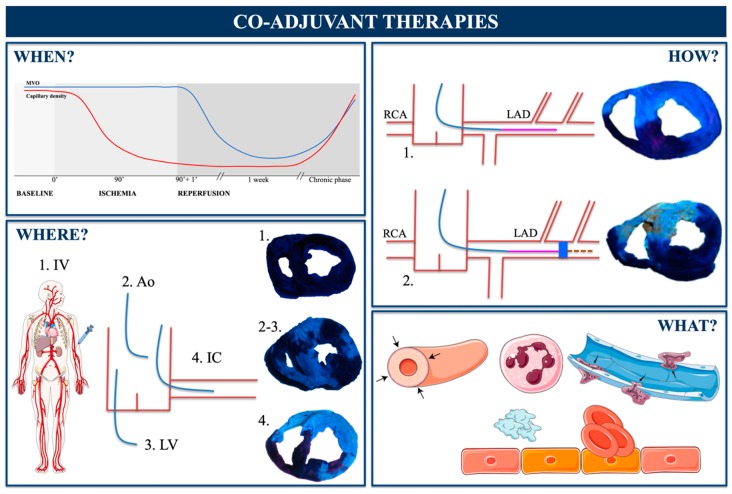
Co-adjuvant therapies beyond reperfusion. Proposed answers to the WH-questions. To optimize the potential effects of any novel co-adjuvant therapies on microvascular obstruction (MVO), the timing (when), the route of administration (where), the technique (how), and the mechanism addressed (what) are crucial. Ao = aorta; IC = intracoronary; IV = intravenous; LAD = left anterior descending; LV = left ventricle; RCA = right coronary artery.

MVO is a highly dynamic process throughout onset, extension, and repair (Figure 6). A thorough comprehension of all its phases is mandatory to better understand how already available clinical strategies can minimize its effects and how knowledge gained from basic research can inspire future opportunities for repair. Eventually, the efficacy of novel approaches will rely not only on “what” therapy (beyond reperfusion) we use, but also on “when”, “where”, and “how”, these co-adjuvant treatments should be administered (Figure 7).

Regarding MVO dynamics, in STEMI patients, extensive MVO detected at first week CMR vanishes a few weeks after reperfusion [8,79,80]. In a swine model of STEMI, macroscopic MVO (Figure 6, blue line) occurs immediately after reperfusion, while capillary density (Figure 6, red line) diminishes during the ischemic phase before reperfusion. Macroscopic and microscopic microvascular damage peak at the subacute phase (one week after reperfusion) and almost completely resolve at the chronic phase (one month after reperfusion). The capacity of swine coronary serum to induce self-protective angiogenesis on an in vitro endothelial coronary cell differentiation assay (Figure 6, green line) is increased very soon after ischemia [10]. This can mediate the complete restoration of microvascular perfusion into the infarcted region one month after reperfusion [8,81].

The loss of small vessels during acute ischemia [10] should be combated in a multidisciplinary manner with logistical measures to optimally reduce the duration of ischemia [2]. The second critical moment for prevention is reperfusion: the invasive and pharmacological management of patients during this brief but crucial period of time needs to be flawless.

Interestingly, the MVO wave exhibits a much slower progression in small vessels (lasting up to several days) than that of necrosis in cardiomyocytes (Figure 6). This offers the opportunity for a potentially longer therapeutic time window to administer possible future co-adjuvant therapies, compared with the scant 4–6 h we have to save myocardium [11].

Along with the dynamics of occurrence, the dynamics of repair are equally important for comprehending MVO pathophysiology and answering the “WH” questions. Unlike ineffective myocardial regeneration, our organism quickly begins pro-angiogenic mechanisms that repair microcirculation loss and contribute to the spontaneous regeneration of the microvasculature that, in general, is successfully completed a few weeks after reperfusion [10] (Figure 6, green line).

Interventional cardiology will probably be crucial in the implementation of a new paradigm that cannot be focused solely on the epicardial artery, but must include increasing attention being placed on preventing, minimizing, and repairing microcirculation damage. For now, we can only speculate on answers to the “when”, “where”, and “how” questions (Figure 7).

The loss of capillary density begins upon ischemia onset and boosts at reperfusion. Thus, the desired pro-angiogenic effect of any co-adjuvant therapy should commence as soon as possible during the reperfusion process, and continue for some weeks until the physiological recovery of the microvasculature is completed (Figure 7). Modern pharmacology and gene therapy technology have been developed that can stimulate pro-angiogenic factors or block anti-angiogenic mediators in a controlled fashion and overcome the challenges of “when”.

Our group explored how the route of administration of the light blue colorant thioflavin-S influences its arrival at the infarcted myocardium [81]. A super-selective release of products into the area at risk (“where” its effect is needed) at the time of reperfusion can be easily achieved by administering potential therapies through the lumen of one of the angioplasty balloons used for primary angioplasty [12] (Figure 7).

A brief transient inflation of the balloon at the point of occlusion simultaneous to the local release of co-adjuvant therapies can permit adequate and selective uptake of products by the infarcted tissue, avoiding the wash-out phenomenon and the unwanted consequences in distal territories or organs [12]. This addresses the “how” question (Figure 7).

Unfortunately, the “what” question remains unanswered and none of the therapies addressed to combat the previously enumerated factors underlying MVO pathophysiology have resulted in consistent clinical benefits in STEMI patients (Table 1). Therefore, a deeper comprehension of MVO pathophysiology is essential for selecting the specific triggering mechanisms (what) implicated in MVO progression. Probably, several of these mechanisms need to be addressed simultaneously. Which of them should be included in such a multifactorial strategy is currently unknown. For instance, the INFUSE-AMI randomized trial evaluated the synergic effect of intracoronary abciximab and aspiration thrombectomy. The combination of both approaches resulted in a lower CMR-derived MVO compared to intracoronary abciximab, pointing out the importance of a multidisciplinary approach to reduce MVO [42].

These days, MVO repair represents a promising but almost unexplored route. The effect of stem cell therapy on MVO has been addressed as a secondary end-point, but, similarly to myocardial repair, basic evidence is scarce and results in patients have been discouraging (Table 1). On the other hand, the molecular regulation of myocardial vascular regeneration is now being meticulously understood and modulation of its key players can become an excellent target. The decisive roles of hypoxia inducible factor-1A (the master factor of pro-angiogenesis), annexin A1 (a neutrophil-derived protein that promotes pro-angiogenic macrophages), and vascular endothelial growth factor-A_165_b (a novel anti-angiogenic factor) have been recently described [10,82,83]. Stimulation of the two former and blockage of the latter illustrate, among others, future therapeutic opportunities to regulate MVO repair.

The current lines of thought herein described emphasize that not only “what” but also “when”, “where”, and “how” therapies are administered can be decisive for achieving the desired goal. Indeed, due to the complex MVO pathophysiology, multi-factorial design trials would be interesting to evaluate the synergistic effect of two interventions at the same time.

Obviously, all these speculations should strictly follow the previously-mentioned steps of translational research from bench to bedside.

## 7. Conclusions

MVO plays a major role in the pathophysiology of STEMI. Its occurrence after coronary reperfusion exerts deleterious structural and clinical consequences.

Late gadolinium-enhancement CMR has become the gold standard of non-invasive imaging techniques for the detection and quantification of microvascular damage after STEMI.

Multiple measures have been investigated as adjuvants to reperfusion aimed at reducing MVO. Nevertheless, the promising results from experimental and preliminary clinical studies have not been adopted in clinical practice. This probably reflects the complex pathophysiology of this phenomenon and the need for a still-to-be-designed comprehensive approach.

In contrast to myocardial regeneration, we must pay special attention to the spontaneous repair of microcirculation after STEMI. An adequate understanding and modulation of this phenomenon could represent an excellent and pragmatic therapeutic opportunity in the future.

Just one century ago, MI was regarded as an entity that necessarily implied the death of patients. In one hundred years, that belief has completely changed; however, MVO remains one of the last hurdles to overcome in completing this success. Important research efforts will be necessary in the basic, pre-clinical, and clinical areas to better understand MVO and to obtain effective therapeutic strategies. Meanwhile, we need to reinforce the already-proven effective measures; namely, education, primary prevention, and universal, timely reperfusion.

## Figures and Tables

**Table 1:** The main clinical studies and clinical trials to evaluate the effect of different therapies in cardiovascular magnetic resonance (CMR)-derived microvascular obstruction (MVO) and major events (death and/or re-infarction), classified according to the pathophysiological factors addressed.

	Design	*n*	CMR-Derived MVO	Major Events	First Author
					[Reference]
1. Patient factors *					
2. Endothelial abnormalities ^†^					
3. Decrease in capillary density ^†^					
4. Ischemia-reperfusion injury					
Post-conditioning	R, OL, PC	78	0	0	Tarantini [26]
	R, OL, PC	101	0		Bodi [27]
	R, OL, PC	50	+		Mewton [28]
	R, OL, PC	68	0		Sörensson [29]
	R, OL, PC	79	0	0	Freixa [30]
	R, OL, PC	102	0		Dwyer [31]
	R, OL, PC	464	0	0	Eitel [32]
Remote ischemic conditioning	R, OL, PC	464	0	0	Eitel [32]
	R, OL, PC	77	0	0	Crimi [33]
	R, OL, PC	83	0		White [34]
Hypothermia	R, OL, PC	18	0		Gotberg [35]
	R, OL, PC	50	0	0	Keeble [36]
	R, OL, PC	120	0	0	Erlinge [37]
	R, OL, PC	101	0		Testori [38]
5. Embolization					
Thrombus aspiration	R, OL, PC	175	+	+	Sardella [39]
	R, SB, PC	154	+	0	De Carlo [40]
	R, OL, PC	40	0	0	Hoole [41]
	R, SB, PC	30	+	0	Ahn [42]
	R, SB, PC	37	0		Carrabba [43]
Bivalirudin	R, OL, PC	78	0		van Geus [44]
	R, OL, PC	51	0		Wohrle [45]
Alteplase	R, DB, PC	440	0	0	McCartney [46]
Erythropoietin	R, OL, PC	50	0	0	Suh [47]
	R, DB, PC	41	0	0	Ludman [48]
	R, OL, PC	102	+	0	Prunier [49]
Clopidogrel	R, OL, PC	198	+	0	Song [50]
Abciximab	R, DB, PC	85	0		Tarantini [51]
	R, SB, PC	169	0		Maehara [52]
	R, SB, Non-PC	138	+		Thiele [53]
Pexelizumab	R, DB, PC	99	0		Patel [54]
Ticagrelor/	Observational	108	0		Vanini [55]
Prasugrel	R, OL, PC	203	0	0	Khan [56]
6. Vasoconstriction					
Adenosine	R, OL, PC	247	0	0	Nazir [57]
	R, DB, PC	201	0		Garcia-Dorado [58]
	R, SB, PC	110	0		Desmet [59]
Nitroprusside	R, OL, PC	247	0	0	Nazir [57]
Nicorandil	R, OL, PC	52	+		Yamada [60]
7. Increase in endothelial permeability ^†^					
8. External compression ^†^					
9. Inflammation					
Atorvastatin	R, OL, PC	37	0	0	Kim [61]
	Non-R, OL, PC	230	0		Marenzi [62]
Metoprolol	R, SB, PC	220	+		Garcia-Prieto [63]
FX06	R, DB, PC	232	0	0	Atar [64]
10. Dynamics and repair					
Stem cell tranfer	R, DB, PC	67	0		Janssens [65]
	R, DB, PC	54	0		Dill [66]
	R, OL, PC	101	0	0	Roncalli [67]
	R, OL, PC	200	0	0	Hirsch [68]
	R, OL, PC	200	0	0	Surder [69]
	R, OL, PC	120	0	0	San Roman [70]
	R, DB, PC	42	0	0	Wohrle [71]
Angiogenesis modulation ^†^					

(+) supports use; (0) no difference between intervention and placebo. Major events are defined as death and/or re-infarction. * Primary prevention (i.e., lifestyle, healthy dietary habits, and smoking habits) and education from childhood are the most effective measures for reducing the incidence of new coronary events, and as a consequence, diminishing the burden of MVO. ^†^ To date, neither clinical studies nor clinical trials specifically addressing these pathophysiological factors are available. B = blinded end-point trial; DB = double-blind; OL = open-label; PC = placebo-controlled; R = randomized; SB = single-blind.
